# Sex Differences in Long-term Outcomes After Group B Streptococcal Infections During Infancy in Denmark and the Netherlands: National Cohort Studies of Neurodevelopmental Impairments and Mortality

**DOI:** 10.1093/cid/ciab822

**Published:** 2021-11-02

**Authors:** Merel N van Kassel, Bronner P Gonçalves, Linde Snoek, Henrik T Sørensen, Merijn W Bijlsma, Joy E Lawn, Erzsébet Horváth-Puhó, GBS Danish and Dutch Collaborative Group for Long-Term Outcomes, Henrik T Sørensen, Erzsébet Horváth-Puhó, Kirstine K Søgaard, Diederik van de Beek, Merijn W Bijlsma, Merel N van Kassel, Linde Snoek, Brechje de Gier, Arie van der Ende, Susan J M Hahné

**Affiliations:** 1 Department of Neurology, Amsterdam Neuroscience, Amsterdam UMC, University of Amsterdam, Amsterdam, The Netherlands; 2 Department of Infectious Disease Epidemiology, London School of Hygiene & Tropical Medicine, London, United Kingdom; 3 Maternal, Adolescent, Reproductive & Child Health Centre, London School of Hygiene & Tropical Medicine, London, United Kingdom; 4 Department of Clinical Epidemiology, Aarhus University, Aarhus N, Denmark; 5 Department of Paediatrics, Amsterdam UMC, University of Amsterdam, Amsterdam, The Netherlands

**Keywords:** *Streptococcus agalactiae*, group B *Streptococcus*, sex differences, effect modification, neurodevelopmental impairments

## Abstract

**Background:**

Male infants have a higher incidence of invasive group B *Streptococcus* disease (iGBS) compared with female infants; however, data on sex differences in mortality and long-term outcomes after iGBS are lacking. We assessed whether a child’s sex influences the effects of iGBS on mortality and risk of neurodevelopmental impairments (NDIs).

**Methods:**

We used Danish and Dutch registry data to conduct a nationwide cohort study of infants with a history of iGBS. A comparison cohort, children without a history of iGBS, was randomly selected and matched on relevant factors. Effect modification by sex was assessed on additive and multiplicative scales.

**Results:**

Our analyses included data from children with a history of iGBS in Denmark (period 1997 -2017; n = 1432) and the Netherlands (2000 -2017; n = 697) and from 21 172 children without iGBS. There was no clear evidence of between-sex heterogeneity in iGBS-associated mortality. Boys had a higher risk of NDI, with evidence for effect modification on additive scale at the age of 5 years for any NDI (relative excess risk due to interaction = 1.28; 95% confidence interval [CI], -0.53 to 3.09 in Denmark and 1.14; 95% CI, -5.13 to 7.41 in the Netherlands). A similar pattern was observed for moderate/severe NDI at age 5 years in Denmark and age 10 years in the Netherlands.

**Conclusion:**

Boys are at higher risk of NDI ; our results suggest this is disproportionally increased in those who develop iGBS. Future studies should investigate mechanisms of this effect modification by sex.

**KEY FINDINGS**

**1. What Is Known and What Is New?**

In this study, we observed that boys with a history of iGBS are at higher risk of neurodevelopmental impairments (NDIs) and more often require special educational support than girls who had iGBS. Importantly, our data provide some evidence that male sex modifies the effect of iGBS on NDI risk on the additive (risk-difference) scale.

**2. What Did We Do and What Did We Find?**

We observed that boys with a history of iGBS are at higher risk of neurodevelopmental impairments (NDIs) and more often require special educational support than girls who had iGBS. Importantly, our data provide some evidence that male sex modifies the effect of iGBS on NDI risk on the additive (risk-difference) scale.

**3. What to Do Now in Programs?**

Our findings on NDI risk after iGBS suggest that even when appropriate treatment is given in the acute phase, extended follow-up care of iGBS survivors might be beneficial to identify NDI early and provide support where needed (eg, educational). The potential effect modification by sex also suggests that this extended follow-up could have higher population-level benefit for boys, who have higher absolute risks of NDI.

**4. What’s Next for Research?**

In low- and middle-income countries, where timely access to care is not always possible, NDI risk after iGBS might be even greater, and studies are needed that follow iGBS survivors in these settings and assess whether factors such as sex influence outcome. Early-intervention longitudinal studies should be designed to determine how these children can best be followed and supported and whether some aspects of clinical conduct should be adapted to account for potential sex differences in risk. Future studies should also investigate mechanisms through which the neurodevelopmental domains might be differentially affected in boys and girls with a history of iGBS.

Group B *Streptococcus* (GBS; *Streptococcus agalactiae*) is an important cause of severe bacterial disease, including sepsis, meningitis, and pneumonia, in neonates and infants worldwide [1, 2]. iGBS during early infancy is responsible for substantial mortality (case fatality risk, 8.4%; 95% confidence interval [CI], 6.6%–10.2%); in survivors, iGBS is associated with long-term NDI, including in the intellectual, motor, vision, and hearing domains [3–5]. Indeed, in a previous analysis, we showed that the risk of NDI and special school education needs approximately doubles after the two common iGBS clinical presentations, sepsis and meningitis [4].

An important next step in GBS clinical research is the identification of factors that influence risk of long-term sequelae, as this would allow improved clinical care with more intensive follow-up targeted at higher-risk groups, and guide future mechanistic studies on the underlying pathogenesis. One factor that has long been known to influence infection risk and outcomes in children is sex [6–8]. For example, there are multiple reports of increased susceptibility to infectious diseases, caused by a wide range of pathogens, in male children compared with female children [9], and sex differences in immune responses have been put forward as a possible explanation [8, 10]. Recently, a meta-analysis also suggested that male infants might be at increased risk of severe neonatal bacterial infections [11], providing additional evidence for earlier findings [7]. Regarding iGBS, sex might be associated with susceptibility as higher incidence has been described in male infants compared with female infants [12–14]. It is noteworthy that sex differences in infectious diseases depend on the studied outcome (eg, incidence, acute severity, sequelae). To our knowledge, data from longitudinal studies with a comparator group on effect modification by sex of mortality and long-term outcomes conditional on the occurrence of iGBS have not been reported.

## AIM 

This paper is part of the series *Every Country,Every Family: Group B Streptococcal Disease Worldwide*. Our aim in this study was to use population-based medical and administrative data collected in Denmark and the Netherlands to assess effect modification [15], that is, variation in effect measure across strata of a background variable, in the context of iGBS outcomes. In particular, we quantified the extent to which a child’s sex modifies the effect of iGBS during early infancy on mortality and long-term NDI risk. In another article in this series, we assessed the influence of prematurity on long-term outcomes after iGBS. Our overarching goal in these 2 articles is to identify clinically relevant factors that might explain variation in the risk of these children to improve clinical care and inform policy.

## METHODS

### Study Design

We conducted nationwide matched cohort studies using Danish population-based medical and administrative registries from 1997–2017 and Dutch medical and administrative databases from 2000–2017, as previously described [4]. iGBS (or exposed) children were defined as having a history of iGBS (GBS sepsis or meningitis) by the age of 89 days. In Denmark, the Danish National Health Service provides tax-supported healthcare, ensuring unfettered access to general practitioners and hospitals for all Danish inhabitants [16]. Children with iGBS were identified from the Medical Birth Registry based on discharge diagnoses (using *International Classification of Diseases, Tenth Revision, Clinical Modification* [ICD-10] codes) registered in the Danish National Patient Registry [17–19]; codes used for the definition of the exposure and outcomes are listed in the Supplementary Materials of a previous publication [4]. This registry contains information on all admissions to Danish nonpsychiatric hospitals since 1977 and on outpatient clinic visits, emergency room visits, and psychiatric hospital visits since 1995. Each hospital discharge or outpatient clinic visit is recorded with 1 primary diagnosis and 1 or more secondary diagnoses classified according to the ICD-8 coding through 1993 and ICD-10 thereafter. In the Netherlands, iGBS children were identified through the Netherlands Reference Laboratory for Bacterial Meningitis; all infants with cerebrospinal fluid and/or blood culture positive for GBS were eligible for inclusion [20, 21]. To each iGBS child, up to 10 children without iGBS were randomly selected and matched on sex, birth year/month, and gestational age categories (<28 weeks, 28–36 weeks, and ≥37 weeks) [22, 23]. The comparison cohorts were defined using the Danish Medical Birth Registry and the Danish Civil Registration System in Denmark and the PeriNed Perinatal Registry and the Municipal Personal Records Database in the Netherlands [22, 23]. Additional information on these cohorts, databases, and codes are reported in a previous study [4].

### Outcome Data

All-cause mortality during the first 89 days and 5 years of life were assessed based on data from the Danish Civil Registration System [18] and the Dutch Municipal Personal Records Database [22]. NDIs were defined differently in the 2 cohorts and have been described previously [4]. Briefly, in Denmark, we obtained information on NDI from the Danish National Patient Registry using ICD-10 codes for mental, behavioral, and nervous system disorders [19]. We assessed motor, hearing, vision, cognitive, and social/behavioral domains, and impairments were categorized by severity (mild/moderate/severe). Note that this registry records information mainly from severe cases; children with milder NDI, only managed and followed in the school system or by general practitioners, are not included in this database. In the Netherlands, special educational support was used as a surrogate marker for NDI. National databases that cover primary school registration and special education were used to identify children who received education in special needs schools (here, considered equivalent to moderate/severe NDI) or who received additional support in regular schools (mild NDI).

### Statistical Analyses

Descriptive analyses and results of statistical models are presented separately for each country. We characterized children with a history of iGBS and children from the comparison cohort by sex according to calendar periods, gestational age (<28, 28–36, 37+ weeks), iGBS clinical syndrome (meningitis, sepsis), and onset of disease (early-onset [aged 0–6 days] and late-onset [aged 7–89 days]). Note that the definition of the age of onset in the Danish cohort was based on the date of hospital admission, and since an unknown proportion of late-onset cases with long-duration hospitalizations could have been misclassified as early-onset cases, data from Denmark are not described by these categories. We calculated mortality risk during the first 3 months and 5 years of life. Mortality rates were estimated per 1000 person-years. Cox proportional hazards regression was used to estimate hazard ratios (HRs), adjusted for gestational age and birth year.

Risks of NDI and special education needs were assessed at ages 5 and 10 years; we included only children followed until at least the corresponding cutoff age. Associations between iGBS and NDI and modification by sex were assessed using logistic regression models to estimate odds ratios (ORs) adjusted for year of birth and gestational age.

We evaluated the extent to which sex modifies the effect of iGBS on mortality and NDI/special education needs [24, 25]. The concept of effect modification relates to variation in effect measures by strata of another variable (see Supplementary Material for a more detailed description of the method). In this analysis, our objectives included assessment of effect modification on both additive and multiplicative scales. Effect modification on the additive scale occurs when differences in rates/risks between exposed and unexposed groups (here, children with a history of iGBS and children without a history of iGBS, respectively) differ by strata of a third variable (here, sex). On the multiplicative scale, modification occurs if relative association measures (ie, odds ratios or hazard ratios) between exposure and outcome vary by strata of a third variable. In conformity with these objectives, our different analyses were estimation of association measures (HRs for mortality and ORs for NDI outcomes) for each combined stratum of sex and iGBS using the common reference category of non-iGBS girls (single reference category analyses); estimation of associations between iGBS and mortality and NDI outcomes among boys and girls (stratified analyses); estimation of effect modification on additive scale by calculating the interaction contrast for mortality using the formula (Mortality rate_iGBS, Boys_ – Mortality rate_IGBS, Girls_) – (Mortality rate_non-iGBS, Boys_ – Mortality rate_non-IGBS, Girls_)) and the relative excess risk due to interaction (RERI) for NDI using the formula OR_iGBS, Boys—ORiGBS, Girls—ORnon-IGBS, Boys_ + 1; and assessment of effect modification on the multiplicative scale by including a product term (sex × iGBS) in the multivariable regression models. Furthermore, in tables in the Results section and Supplementary Material, we also present the attributable proportion, which here was calculated as: (OR_iGBS, Boys—ORiGBS, Girls—ORnon-IGBS, Boys_ + 1)/(OR_iGBS, Boys_).

In addition to the analyses described above, we also performed secondary analyses that used cohort children with no history of iGBS and who were not matched on gestational age as a comparison. In these secondary analyses that used a cohort of children with no, prematurity status was not included as a predictor.

Analyses were conducted using SAS version 9.4 (Denmark) and SPSS Statistics version 25.0 and STATA version 16 (the Netherlands). The Danish Data Protection Agency approved the study. In the Netherlands, the study protocol was submitted to the Centre for Clinical Expertise at the National Institute for Public Health and the Environment. It was exempted from further approval by an ethical research committee, according to Dutch law for medical research involving human subjects.

## RESULTS

### Clinical Characteristics

**Figure 1.**
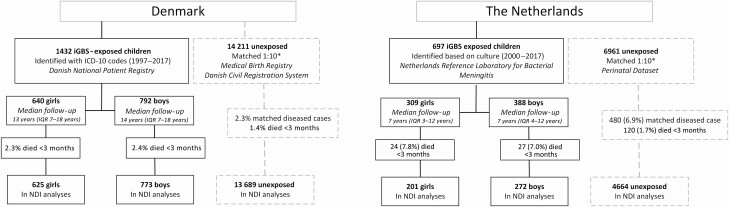
Clinical characteristics. Abbreviations: ICD-10, *International Classification of Diseases, Tenth Revision, Clinical Modification*; iGBS, invasive group B *Streptococcus* disease; IQR, interquartile range; NDI, neurodevelopmental impairment.

We identified 2129 children with a history of iGBS, of whom 1180 (55%) were boys and 949 (45%) were girls (Table 1, Figure 1). A total of 1763 (83%) iGBS children had a history of GBS sepsis and 366 (17%) had a history of meningitis. Most iGBS children in the Dutch cohort, boys or girls, had early-onset disease (445, 63.8%). A total of 487 (22.9%) iGBS children were preterm (born before 37 weeks of gestation); this percentage did not differ between boys and girls.

**Table 1.** Characteristics of Girls and Boys With Invasive Group B *Streptococcus* Disease and Members of Matched Comparison Cohorts in Denmark and the Netherlands

	Denmark				The Netherlands			
	Girls		Boys		Girls		Boys	
Baseline variables	GBS–	GBS+	GBS–	GBS+	GBS–	GBS+	GBS–	GBS+
Total^a^	6342	640	7869	792	3081	309	3880	388
Study period								
1997–1999	1283 (20.2)	129 (20.2)	1720 (21.9)	174 (22.0)				
2000–2005	2019 (31.8)	204 (31.9)	2577 (32.7)	258 (32.6)	704 (22.8)	71 (23.0)	920 (23.7)	92 (23.7)
2006–2011	1772 (27.9)	179 (28.0)	1849 (23.5)	186 (23.5)	867 (28.1)	87 (28.2)	1230 (31.7)	123 (31.7)
2012–2017	1268 (20.0)	128 (20.0)	1723 (21.9)	174 (22.0)	1510 (49.0)	151 (48.9)	1730 (44.6)	173 (44.6)
Gestational age, weeks								
<28	202 (3.2)	26 (4.1)	189 (2.4)	24 (3.0)	151 (4.9)	16 (5.2)	140 (3.6)	14 (3.6)
28–36	1140 (18.0)	114 (17.8)	1420 (18.0)	142 (17.9)	700 (22.7)	70 (22.7)	810 (20.9)	81 (20.9)
37+	5000 (78.8)	500 (78.1)	6260 (79.6)	626 (79.0)	2230 (72.4)	223 (72.2)	2930 (75.5)	293 (75.5)
Syndrome								
Meningitis	-	89 (13.9)	-	79 (10.0)	-	88 (28.5)	-	110 (28.4)
Sepsis	-	551 (86.1)	-	713 (90.0)	-	221 (71.5)	-	278 (71.6)
Onset of disease^b^								
Early onset (<1 week)	-	-	-	-	-	209 (67.6)	-	236 (60.8)
Late onset (>1 week)	-	-	-	-	-	100 (32.4)	-	152 (39.2)

Numbers within parenthesis represent percentages.

Abbreviation: GBS, group B *Streptococcus* disease.

^a^To each invasive GBS child, up to 10 children without iGBS were randomly selected and matched on sex, birth year/month, and gestational age categories (<28 weeks, 28–36 weeks, and ≥37 weeks).

^b^In the Netherlands, the first reported date of illness, primarily, the first date a culture was taken (97.4%), was used to calculate age of onset. We do not report date of onset for Denmark.

### Mortality

In Denmark, 2.3% (95% CI, 1.4%–3.9%) of girls and 2.4% (95% CI, 1.5%–3.7%) of boys with iGBS disease died within 3 months after birth compared with 1.8% (95% CI, 1.5%–2.1%) of girls and 1.5% (95% CI, 1.3%–1.8%) of boys from the comparison cohort. Mortality rates up to age 5 years (ie, 0–5 years) were not different in both GBS-exposed (sepsis and meningitis combined) boys and girls compared with their respective non-iGBS matches (HR, 0.97; 95% CI, 0.60–1.57 among boys and HR, 1.00; 95% CI, 0.62–1.62 among girls; Table 2). However, a higher 5-year mortality was observed in girls with a history of GBS meningitis compared with their non-iGBS matches (HR, 6.20; 95% CI, 2.13–18.06; Supplementary Table 1), while in boys with a history of GBS meningitis, there was only limited evidence of higher mortality (HR, 2.39; 95% CI, 0.62–9.25).

**Table 2.** Effect Modification by Sex of the Association Between Invasive Group B *Streptococcus* Disease and Mortality.

		Single Reference Category Analyses		Stratified Analyses	
Cohorts	Mortality Rate per 1000 Person-Years	HR (95% CI) for iGBS Using a Common Reference Group	Effect Modification on Additive Scale (Interaction Contrast [95% CI]); AP [%]	HR (95% CI) for iGBS Within Strata of Sex	Effect Modification on Multiplicative Scale (P Value of GBS × Sex)
Denmark					
0–89 days					
GBS–/Girls	73.7 (60.1–87.4)	1.00 (reference)		1.00 (reference)	
GBS+/Girls	98.1 (48.4–147.7)	0.87 (0.51–1.49)		0.89 (0.52–1.53)	
GBS–/Boys	63.5 (52.1–74.9)	1.04 (0.81–1.35)		1.00 (reference)	
GBS+/Boys	100.6 (55.4–145.9)	1.16 (0.71–1.88)	*12.8 (–56.7* to *82.2); 12.7%*	1.08 (0.67–1.76)	*P* = .52
0–5 years					
GBS–/Girls	4.4 (3.7–5.2)	1.00 (reference)		1.00 (reference)	
GBS+/Girls	6.5 (3.6–9.4)	0.97 (0.60–1.57)		1.00 (0.62–1.62)	
GBS–/Boys	3.8 (3.1–4.4)	1.01 (0.80–1.29)		1.00 (reference)	
GBS+/Boys	5.3 (2.9–7.6)	1.01 (0.62–1.63)	*–0.6 (–4.5* to *3.3); NA*	0.97 (0.60–1.57)	*P* = .90
The Netherlands					
0–89 days					
GBS–/Girls	71.7 (52.4–91.0)	1.00 (reference)		1.00 (reference)	
GBS+/Girls	340.7 (204.4–477.0)	4.33 (2.67–7.02)		4.33 (2.67–7.01)	
GBS–/Boys	72.0 (54.8–89.3)	1.24 (0.87–1.79)		1.00 (reference)	
GBS+/Boys	302.7 (188.5–416.8)	4.76 (2.99–7.57)	*–38.3 (–218.0* to *141.4); NA*	3.82 (2.44–5.97)	*P* = .71
0–5 years					
GBS–/Girls	4.6 (3.4–5.8)	1.00 (reference)		1.00 (reference)	
GBS+/Girls	20.2 (12.1–28.3)	3.81 (2.37–6.14)		3.81 (2.36–6.13)	
GBS–/Boys	4.6 (3.6–5.7)	1.17 (0.83–1.65)		1.00 (reference)	
GBS+/Boys	18.8 (12.0–25.7)	4.85 (3.10–7.58)	*–1.4 (–12.1* to *9.3); NA*	4.14 (2.70–6.36)	*P* = .80

HRs are adjusted for matching variables (birth year, gestational age). Due to absence of cases in the extreme preterm age category (<28 weeks), the preterm age categories were merged (<37 weeks) for adjustment purposes.

Abbreviations: AP, attributable proportion; CI, confidence interval; HR, hazard ratio; iGBS, invasive group B *Streptococcus* disease; NA, not applicable.

In the Netherlands, 8.1% (95% CI, 5.3%–11.4%) of girls and 7.2% (95% CI, 4.8%–10.0%) of boys with iGBS disease died within 3 months after birth compared with 1.7% (95% CI, 1.3%–2.3%) of girls and 1.7% (95% CI, 1.4%–2.2%) of boys from the comparison cohort. Mortality rates by age of 5 years were higher in boys and girls with a history of iGBS, both sepsis and meningitis, compared with their unexposed matches: HR, 3.81; 95% CI, 2.36–6.13 for iGBS among girls and HR, 4.14; 95% CI, 2.70–6.36 for iGBS among boys (Table 2). In the Netherlands, boys who developed GBS meningitis had considerably higher mortality rates in the first 3 months of life compared with iGBS girls and non-iGBS boys (Supplementary Table 1).

In both countries, there was only limited or no evidence of effect modification by sex for mortality as implied by interaction contrast analyses and by results of Cox regressions, presented in Table 2 (single reference category analyses). However, only a limited number of events (85 deaths in iGBS children and 322 in the comparison cohort) were analyzed.

### Neurodevelopmental Impairments and Educational Support

**Figure 2.**
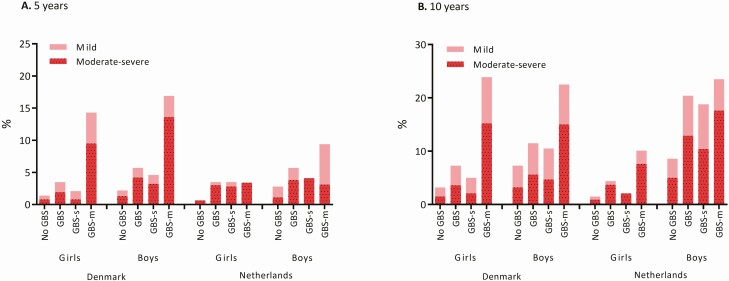
Neurodevelopmental impairments and educational support. Abbreviations: GBS, group B *Streptococcus*; GBS-m, GBS meningitis; GBS-s, GBS sepsis.

In Denmark, 3.5% of iGBS girls and 5.7% of iGBS boys had NDI of any severity by the age of 5 years compared with 1.4% of non-iGBS girls (OR, 2.35; 95% CI, 1.40–3.94) and 2.2% of non-iGBS boys (OR, 2.54; 95% CI, 1.74–3.70; Figure 2, Table 3). In the Netherlands, 3.5% of iGBS girls and 4.4% of iGBS boys received some form of special education by the age of 5 years compared with 0.7% (OR, 5.29; 95% CI, 2.03–13.77) and 1.5% (OR, 3.08; 95% CI, 1.58–5.98) of the respective comparison cohorts (Figure 2, Table 3).

**Table 3.** Effect Modification by Sex of Neurodevelopmental Impairment Outcome After Invasive Group B *Streptococcus* Disease

		Single Reference Category Analyses		Stratified Analyses	
Cohorts	Neurodevelopmental Impairment	OR (95% CI) for iGBS Using a Common Reference Group	Effect Modification on Additive Scale (Relative Excess Risk Due to Interaction [95% CI] and AP [%])	OR (95% CI) for iGBS Within Strata of Sex	Effect Modification on Multiplicative Scale (P Value of GBS × Sex)
Denmark					
5 years					
Any					
GBS–/Girls	1.4 (1.1–1.8)	1.00 (reference)		1.00 (reference)	
GBS+/Girls	3.5 (2.2–5.5)	2.30 (1.37–3.87)		2.35 (1.40–3.94)	
GBS–/Boys	2.2 (1.9–2.6)	1.66 (1.25–2.21)		1.00 (reference)	
GBS+/Boys	5.7 (4.1–7.8)	4.24 (2.82–6.37)	*1.28 (–0.53* to *3.09); 30.2%*	2.54 (1.74–3.70)	*P* = .75
Moderate–severe					
GBS–/Girls	0.8 (0.6–1.1)	1.00 (reference)		1.00 (reference)	
GBS+/Girls	1.9 (0.9–3.4)	2.08 (1.03–4.21)		2.11 (1.04–4.29)	
GBS–/Boys	1.3 (1.0–1.6)	1.73 (1.19–2.51)		1.00 (reference)	
GBS+/Boys	4.2 (2.8–6.0)	5.50 (3.35–9.04)	2.70 (0.09 to 5.30); 49.1%	3.18 (2.03–4.98)	*P* = .32
10 years					
Any					
GBS–/Girls	3.2 (2.6–3.8)	1.00 (reference)		1.00 (reference)	
GBS+/Girls	7.3 (4.9–10.3)	2.32 (1.51–3.57)		2.33 (1.52–3.58)	
GBS–/Boys	7.3 (6.6–8.0)	2.45 (1.98–3.03)		1.00 (reference)	
GBS+/Boys	11.5 (8.8–14.7)	3.97 (2.84–5.54)	*0.20 (–1.27* to *1.66); 5.0%*	1.62 (1.20–2.19)	*P* = .18
Moderate–severe					
GBS–/Girls	1.5 (1.1–1.9)	1.00 (reference)		1.00 (reference)	
GBS+/Girls	3.6 (2.0–6.0)	2.44 (1.34–4.44)		2.47 (1.35–4.51)	
GBS–/Boys	3.2 (2.8–3.8)	2.31 (1.69–3.16)		1.00 (reference)	
GBS+/Boys	5.6 (3.7–8.0)	3.93 (2.45–6.31)	0.18 (–1.88 to 2.25); 4.6%	1.70 (1.11–2.59)	*P* = .34
The Netherlands					
5 years					
Any					
GBS–/Girls	0.7% (0.4–1.1)	1.00 (reference)		1.00 (reference)	
GBS+/Girls	3.5% (1.4–7.0)	5.28 (2.06–13.50)		5.29 (2.03–13.77)	
GBS–/Boys	1.5% (1.0–2.0)	2.61 (1.38–4.93)		1.00 (reference)	
GBS+/Boys	4.4% (2.3–7.6)	8.03 (3.59–17.97)	1.14 (–5.13 to 7.41); 14.2%	3.08 (1.58–5.98)	*P* = .36
Moderate–severe					
GBS–/Girls	0.6% (0.3–1.1)	1.00 (reference)		1.00 (reference)	
GBS+/Girls	3.0% (1.1–6.4)	4.85 (1.78–13.19)		4.86 (1.75–13.50)	
GBS–/Boys	0.9% (0.6–1.4)	1.83 (0.91–3.67)		1.00 (reference)	
GBS+/Boys	3.7% (1.8–6.7)	7.32 (3.09–17.31)	1.64 (–4.59 to 7.87); 22%	4.00 (1.89–8.46)	*P* = .76
10 years					
Any					
GBS–/Girls	2.8% (1.9–4.0)	1.00 (reference)		1.00 (reference)	
GBS+/Girls	5.7% (2.1–12.0)	2.08 (0.84–5.16)		2.05 (0.82–5.13)	
GBS–/Boys	8.6% (7.3–10.2)	3.50 (2.31–5.29)		1.00 (reference)	
GBS+/Boys	20.4% (14.2–27.8)	9.54 (5.50–16.55)	*4.97 (0.43* to *9.51); 52.1%*	2.73 (1.75–4.25)	*P* = .60
Moderate–severe					
GBS–/Girls	1.1% (0.5–1.9)	1.00 (reference)		1.00 (reference)	
GBS+/Girls	3.8% (1.1–9.5)	3.77 (1.17–12.13)		3.86 (1.18–12.63)	
GBS–/Boys	5.0% (3.9–6.2)	5.18 (2.73–9.83)		1.00 (reference)	
GBS+/Boys	12.9% (8.0–19.5)	15.01 (6.94–32.47)	7.06 (–2.05 to 16.16); 47.0%	2.88 (1.68–4.94)	*P* = .69

ORs are adjusted for matching variables (birth year, gestational age).

Abbreviation: AP, attributable proportion; CI, confidence interval; iGBS, invasive group B *Streptococcus* disease; OR, odds ratio.

In both countries, a higher risk of NDI was observed in non-iGBS boys, iGBS boys, and iGBS girls compared with the common reference category of non-iGBS girls. There was evidence for effect modification on the additive scale (ie, risk difference scale) in both Denmark and the Netherlands at age 5 years for NDI (RERI, 1.28; 95% CI, –0.53 to 3.09 and RERI, 1.14; 95% CI, –5.13 to 7.41, respectively). At the age of 10 years, effect modification on the additive scale was also observed in the Netherlands (RERI, 4.97; 95% CI, 0.43 to 9.51). Our results also provide evidence for effect modification on the additive scale at the age of 5 years for moderate and severe NDI (RERI, 2.70; 95% CI, 0.09 to 5.30 in Denmark and RERI, 1.64; 95% CI, –4.59 to 7.87 in the Netherlands) and at 10 years of age in the Netherlands (RERI, 7.06; 95% CI, –2.05 to 16.16). Similar patterns of effect modification were observed in analyses of GBS sepsis and GBS meningitis (Supplementary Table 2). When comparing the exposed cohort with a nongestational age-matched comparison cohort (Supplementary Table 3), similar results were obtained in Denmark. In the Netherlands, there was a reduction in the effect modification on the additive scale and evidence for effect modification on the multiplicative scale. In Denmark, domain-specific data were suggestive of additive interaction in motor domain–related NDI (Supplementary Table 4).

In both countries, there was no robust evidence of effect modification by sex for NDI outcomes on the multiplicative scale (Table 3), that is, relative measures of association (odds ratio) did not differ substantially between boys and girls.

## Discussion

Data on sex differences in relation to iGBS, a major cause of severe infection in infants, were lacking. Our results confirm and refine previous findings that show that iGBS is associated with significant increase in NDI risk in survivors [4]. Indeed, in the large Danish cohort, we observed that boys are at higher risk of NDI, whether exposed or not to iGBS, and that this risk is even higher in iGBS survivors. Furthermore, the Dutch results indicate that boys require support at school more often than girls, in particular, boys with a history of iGBS. Our findings represent a step toward better NDI risk stratification for iGBS survivors and imply that a low threshold for follow-up care of iGBS survivors, especially boys, is needed. From a public health perspective, effect modification by sex on the additive (risk difference) scale implies more preventable cases of GBS-related NDI are affecting boys, which should be considered in policy design.

**Figure 3.**
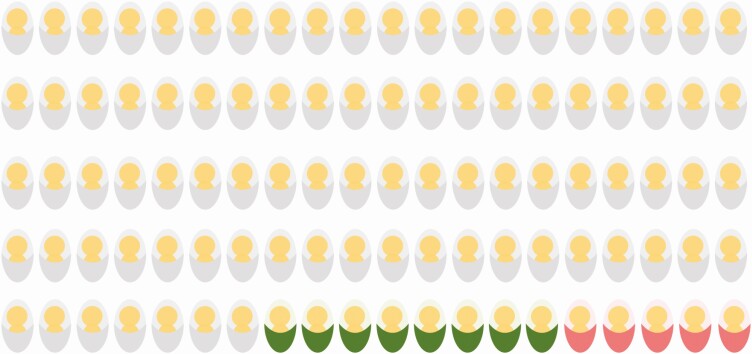
Assessing effect modification. Hypothetical example of long-term outcome in 100 Dutch boys who survived invasive GBS disease, 13 of whom will have moderate or severe NDI after iGBS. Five of these thirteen will be linked to effect modification by sex.

By assessing effect modification, we aimed to provide relevant information to public health professionals as effect modification on the additive scale informs questions such as, would preventative measures reduce more cases if applied to boys or girls? We also assessed modification on the multiplicative scale that, in this case, would be relevant for pediatric practice as it quantifies whether relative associations between iGBS and NDI risk depend on the child’s sex. Our data provide evidence for effect modification of the risk of NDI after iGBS by sex, on the additive scale, in both countries. Indeed, boys have a higher risk of NDI and educational support needs compared with girls, even when unexposed to iGBS, which is consistent with the previous observation that, in general, boys are performing worse at school and more often require extra support at school compared with girls [26, 27]. While in Denmark this effect modification was only present at the age of 5 years, in the Netherlands it was still evident by the age of 10 years; for example, in the Dutch cohort, of 100 boys with history of iGBS, approximately 13 will be diagnosed with moderate/severe NDI (special education needs), with 5 of these linked to effect modification by sex (Figure 3). It is possible that this difference in pattern between the countries is related to the different definitions of NDI outcome used in the 2 countries: in Denmark, the NDI outcome definition was based on ICD-10 codes for mental, behavioral, and nervous system disorders, which implies more severe handicaps were more likely to be diagnosed at an early age compared with milder forms of impairment. In the Netherlands, the requirement for extra support at school, which could be associated with undiagnosed impairment of presumably lesser severity early in life, was used as a functional outcome of neurodevelopment. If sex not only influences the magnitude of the association between iGBS exposure and NDI but also accelerates the development of adverse outcomes (eg, if NDI is diagnosed at earlier ages in boys), then it is possible that the degree of effect modification might vary with age.

Although there are known differences between boys and girls in fatality risks for other infections, mortality during or after iGBS was not substantially affected by sex in Denmark and the Netherlands. However, as mentioned in the Results section, the relatively small number of death events in our cohorts might not have been sufficient to robustly quantify effect modification in this outcome. Systematic reanalysis of published data from other settings, including Sub-Saharan Africa, where most deaths are estimated to occur, might provide additional insights into this question [5, 28].

Our study has strengths including the large sample size of iGBS children and children from the comparison cohort. This is essential given that the risk of some of the outcomes studied is low; more than 1500 children with iGBS were included in analyses of NDI outcomes in Denmark and the Netherlands. Long-term follow-up data are also a crucial component in epidemiological studies that assess the impact of neonatal clinical conditions on development. As suggested by our data from the Netherlands, some important patterns might only be observable at older ages (eg, effect modification by sex on the additive scale at age 10 years). Furthermore, the clinical information available that allowed categorization of iGBS episodes either as sepsis or meningitis confirmed that both syndromes are associated with long-term adverse outcomes in iGBS survivors and provides information on syndrome-specific effect modification by sex. However, the study also has limitations. For example, by using ICD-10 codes rather than actively assessing NDI in enrolled children, it is possible that the risk of mild NDI after iGBS disease, which contributes to the risk of “any” severity NDI, was underestimated in Denmark and/or that primarily severe cases were identifiable using this approach. We also cannot rule out that if NDI in boys more often leads to externalizing behaviour compared with NDI in girls [29, 30], then impairment in boys is more readily diagnosable. Furthermore, in the Netherlands, the iGBS case identification required culture confirmation, which is known to have limited sensitivity, especially after antibiotic use initiation. If culture positivity is associated with acute and long-term outcomes after iGBS, the association between iGBS and special education needs might be different in the overall population of iGBS cases. For a secondary analysis that did not match or adjust for gestational age, while effect modification results in Denmark did not change considerably, in the Netherlands, additive scale effect modification for the NDI outcome was reduced, and multiplicative scale effect modification increased. It is likely that confounding might have contributed to the change observed in the unadjusted analysis, and it is important that future epidemiological studies disentangle causal links between maternal GBS colonization, invasive GBS disease, and preterm births. Finally, as with any observational study, it is not possible to rule out the existence of unmeasured confounders.

The results of this study confirm the need for continued follow-up during early childhood for children who developed iGBS, regardless of the clinical presentation. While NDI risk after iGBS has been previously described [3], the quantification of this risk by demographic and clinical characteristics using long-term cohorts has not been undertaken. Identification of factors that can predict long-term outcomes after an iGBS episode during infancy is important to improve medical care and to provide support where needed (eg, at school). Our findings show that boys are at higher risk of long-term sequelae after iGBS, but the mechanism behind this difference and whether it relates to the higher risk of NDI in boys in the general population are unknown. It is also unclear whether the same pattern of sex heterogeneity occurs when assessing the long-term consequences of other severe bacterial infections during infancy. We are planning to investigate this question, expanding our approach to other clinically important pathogens. Importantly, this study presents results of 2 European high-income countries and might not reflect risks and sex differences in low- and middle-income countries. Indeed, global epidemiological estimates of GBS disease suggest that most of the iGBS burden occurs in Asia and Africa in terms of numbers of early- and late-onset cases. In these regions, both mortality associated with acute episodes and long-term risk of NDI after iGBS are likely higher than in Europe due to, in general, more limited access to care.

## Supplementary Data

Supplementary materials are available at *Clinical Infectious Diseases* online. Consisting of data provided by the authors to benefit the reader, the posted materials are not copyedited and are the sole responsibility of the authors, so questions or comments should be addressed to the corresponding author.

ciab822_suppl_Supplementary_Figure_S1

ciab822_suppl_Supplementary_Material

## Notes

Collaborative group members are listed in the Notes section.

## Notes

***Group B Streptococcus Danish and Dutch Collaborative Group for Long-Term Outcomes*:** Henrik T Sørensen (Department of Clinical Epidemiology, Aarhus University, Aarhus N, Denmark), Erzsébet Horváth-Puhó (Department of Clinical Epidemiology, Aarhus University, Aarhus N, Denmark), Kirstine K Søgaard (Department of Clinical Epidemiology, Aarhus University, Aarhus N, Denmark; Department of Clinical Microbiology, Aalborg University Hospital, Aalborg, Denmark), Diederik van de Beek (Department of Neurology, Amsterdam Neuroscience, Amsterdam UMC, University of Amsterdam, Amsterdam, Netherlands), Merijn W Bijlsma (Department of Neurology, Amsterdam Neuroscience, Amsterdam UMC, University of Amsterdam, Amsterdam, Netherlands), Merel N van Kassel (Department of Neurology, Amsterdam Neuroscience, Amsterdam UMC, University of Amsterdam, Amsterdam, Netherlands), Linde Snoek (Department of Neurology, Amsterdam Neuroscience, Amsterdam UMC, University of Amsterdam, Amsterdam, Netherlands), Brechje de Gier (Centre for Infectious Disease Control, National Institute for Public Health and the Environment, Bilthoven, Netherlands), Arie van der Ende (Department of Medical Microbiology and Infection Prevention and the Netherlands Reference Laboratory for Bacterial Meningitis, Amsterdam UMC, University of Amsterdam, Amsterdam, Netherlands), and Susan J M Hahné (Centre for Infectious Disease Control, National Institute for Public Health and the Environment, Bilthoven, Netherlands)

***Author contributions.*** J. E. L. conceptualized the group B *Streptococcus* study and the idea for sex-specific risk. All site teams contributed to the study design and protocol, accessed the data, and performed the analyses. E. H.-P. (Danish data) and M. N. v. K. (Dutch data) planned and performed the analyses. B. P. G. provided statistical oversight. M. N. v. K., E. H.-P., and B. P. G. drafted the manuscript. All authors reviewed and helped to revise the manuscript and reviewed and agreed on the final version.

***Supplement sponsorship.*** This supplement is sponsored by Bill & Melinda Gates Foundation.

***Acknowledgments.*** We thank PeriNed and Statistics Netherlands for their cooperation and for making their valuable data available for this study.

***Disclaimer.*** The funders had no role in study design, data collection and analysis, decision to publish, or preparation of the manuscript.

***Financial support.*** This work was supported by a grant (INV-009018) from the Bill & Melinda Gates Foundation to the London School of Hygiene & Tropical Medicine (Principal Investigator, Joy Lawn).” Without further mention of the sub-awards. We would suggest doing the same for this manuscript. However, If CID decides it is necessary to name the sub-awards, then change 10.16 “subawards from the LSHTM to M. Bijlsma (and D. van de Beek) Amsterdam UMC the Netherlands” to “subawards from the LSHTM to M. Bijlsma (and D. van de Beek) Amsterdam UMC the Netherlands (AMC ZM0918)”.

***Potential conflicts of interest.*** All remaining authors: No reported conflicts of interest. All authors have submitted the ICMJE Form for Disclosure of Potential Conflicts of Interest. Conflicts that the editors consider relevant to the content of the manuscript have been disclosed.
