# Supplementary figures and images for: Sex Differences in Long-term Outcomes After Group B Streptococcal Infections During Infancy in Denmark and the Netherlands: National Cohort Studies of Neurodevelopmental Impairments and Mortality
Source: Clin Infect Dis. 2021 Nov 2;74(Suppl 1):S54–63. doi: 10.1093/cid/ciab822 (PMC8775649; doi:10.1093/cid/ciab822)

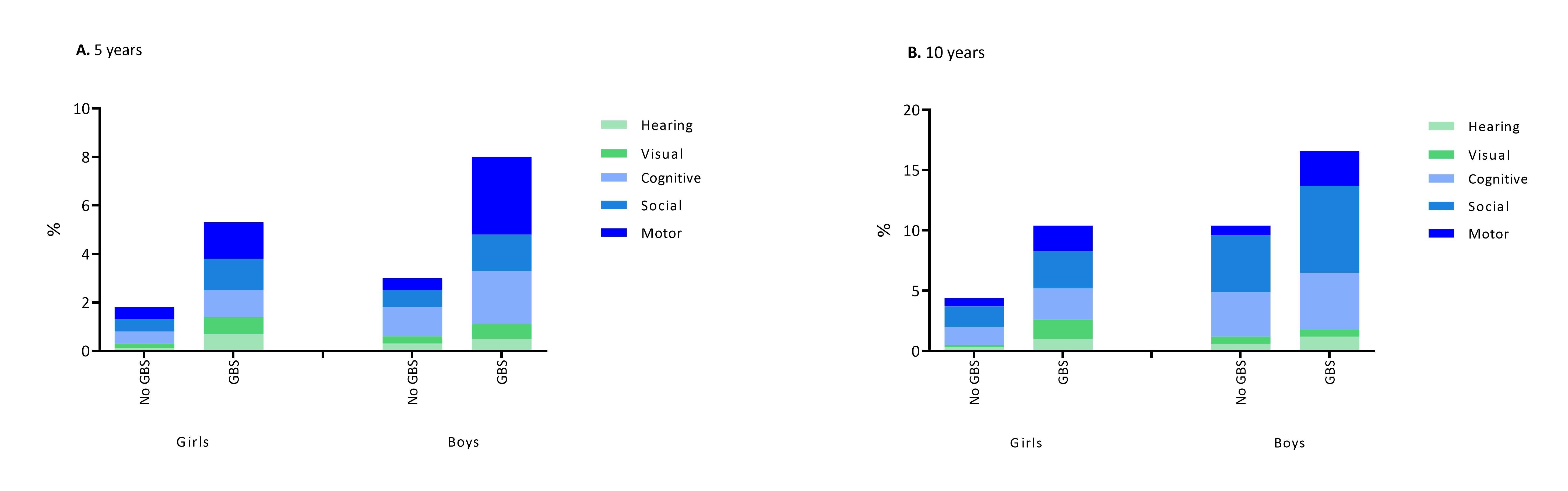

Supplement: ciab822_suppl_Supplementary_Figure_S1 [file ciab822_suppl_supplementary_figure_s1.jpeg]
