# Supplementary material for: Sex Differences in Long-term Outcomes After Group B Streptococcal Infections During Infancy in Denmark and the Netherlands: National Cohort Studies of Neurodevelopmental Impairments and Mortality
Source: Clin Infect Dis. 2021 Nov 2;74(Suppl 1):S54–63. doi: 10.1093/cid/ciab822 (PMC8775649; doi:10.1093/cid/ciab822)
Supplement: ciab822_suppl_Supplementary_Material [file ciab822_suppl_supplementary_material.docx]

**SUPPLEMENTARY MATERIAL**

**Supplement Title**: Every Country, Every woman, Every Child; Group B Streptococcal Disease Worldwide

**Paper Title**: Sex differences in long-term outcomes after Group B streptococcal infections during infancy in Denmark and the Netherlands: national cohort studies of neurodevelopmental impairments and mortality

**Short title**: Sex differences in outcomes after GBS infections

**Authors:** Merel N. van Kassel^1^*, Bronner P. Gonçalves^2,3^*, Linde L. Snoek^1^, Henrik T. Sørensen^4^, Merijn W. Bijlsma^1,5 #^, Joy E. Lawn^2,3#^, Erzsébet Horváth-Puhó^4#^ # on behalf of the GBS Danish and Dutch collaborative group for long term outcomes

*Corresponding author

**Affiliations**

1 Department of Neurology, Amsterdam Neuroscience, Amsterdam UMC, University of Amsterdam, Amsterdam, Netherlands

2 Department of Infectious Disease Epidemiology, London School of Hygiene & Tropical Medicine, London, United Kingdom

3 Maternal, Adolescent, Reproductive & Child Health (MARCH) Centre, London School of Hygiene & Tropical Medicine, London, United Kingdom

4 Department of Clinical Epidemiology, Aarhus University, Aarhus N, Denmark

5 Department of Paediatrics, Amsterdam UMC, University of Amsterdam, Amsterdam, Netherlands

**Contents**

[Additional information on national databases 3](#_Toc81569564)

[Supplementary Methods 6](#_Toc81569565)

[Supplementary tables 8](#_Toc81569566)

[Supplementary Table 1A. Effect modification by sex of mortality after GBS sepsis 1](#_Toc81569567)

[Supplementary Table 1B. Effect modification by sex of mortality after GBS meningitis 1](#_Toc81569568)

[Supplementary Table 2A. Effect modification by sex of NDI outcome after GBS sepsis 1](#_Toc81569569)

[Supplementary Table 2B. Effect modification by sex of NDI outcome after GBS meningitis 3](#_Toc81569570)

[Supplementary Table 4. Effect modification by sex of domain-specific NDI outcomes after invasive GBS disease in Denmark 3](#_Toc81569571)

[References 7](#_Toc81569572)

# Additional information on national databases

In this section below, which was adapted from the Supplementary Appendix of our previous publication on this study population^1^ (<https://ars.els-cdn.com/content/image/1-s2.0-S2352464221000225-mmc3.pdf>), we provide additional information on the national databases that were used both to assess exposure (history of invasive GBS disease [iGBS]) and outcomes (mortality, neurodevelopment impairments).

***Denmark***

The Danish National Health Service provides tax-supported health care, ensuring unfettered access to general practitioners and hospitals for all Danish inhabitants.^2^ Accurate linkage of all registries is possible in Denmark at the individual level using the unique Civil Personal Register (CPR) number assigned to each Danish citizen at birth and to residents upon immigration.^3^

In our analyses, the following databases were used:

- Danish Civil Registration System: This database is an administrative register established in 1968. It contains individual-level information on all persons residing in Denmark. Civil Registration System provides daily updates on vital statistics, including dates of birth, migration, emigration, and death. Upon registration in the Civil Registration System, each person receives a unique ten-digit identification number (CPR number), which allows for cost-effective and unambiguous individual-level record linkage of different Danish registers. The CPR number is used in all Danish administrative and medical registers containing birth-related, vital status, clinical, healthcare utilization, and income data.^3^
- Danish Medical Birth Registry: This is a key component of the Danish health information system. The Medical Birth Registry permits the health of pregnant women and their offspring to be monitored. The register was established in 1973 based on paper birth forms and includes prospectively collected data on all deliveries in Denmark. Major changes in the construction and content of the Medical Birth Registry were implemented in 1997 when the electronic registration of births replaced paper forms. The Medical Birth Registry contains information on the index pregnancy (including CPR numbers of the parents), pregnancy-related characteristics of the mother (e.g. parity, pregnancy-related complications), details of the delivery (e.g. date of delivery, caesarean section), and outcome characteristics of the newborn (e.g. gestational age, Apgar score, birth weight).^4^
- Danish National Patient Registry: This registry is one of the world’s oldest nationwide hospital registries and is used extensively for research.^5-7^ The National Patient Registry contains information recorded on all admissions to Danish non-psychiatric hospitals since 1977 and on outpatient clinic visits and emergency room visits since 1995. Each hospital discharge or outpatient clinic visit is recorded in the National Patient Registry with one primary diagnosis and one or more secondary diagnoses classified according to the International Classification of Diseases, Eighth Revision (ICD-8) through 1993 and Tenth Revision (ICD-10) thereafter. The National Patient Registry contains information also on examinations, certain inpatient medical treatments, and surgical procedures.^8^
- Danish Psychiatric Central Research Registry: This database contains information on every psychiatric admission from 1969 until the present. In 1995, data on outpatient clinic treatment and emergency room contacts were added and the Psychiatric Central Research Registry became an integrated component of the National Patient Registry.^9^ The register contains the CPR-number, dates of any admission and discharge or start and end of any outpatient treatment including emergency room visits; all diagnoses; type of referral; place of treatment with identification of the specific department; municipality of residence; and mode of admission (acute or planned).

***The Netherlands***

The Dutch Healthcare System is a social welfare-based system, with mandatory health care insurance for all residents aged 18 years or older. The System provides a standard, nearly comprehensive, benefit package specified by law.^13^

The following databases were used in this cohort study:

- The Netherlands Reference Laboratory for Bacterial Meningitis database: The Reference Laboratory started collecting *Neisseria meningitides* isolates in 1959 and of other bacteria causing meningitis from 1975 onwards, covering approximately 90 percent of all isolates cultured from CSF of patients with a (suspected) bacterial meningitis in the Netherlands.^14,15^ Additionally, microbiology laboratories in the Netherlands also send blood isolates of infants with invasive GBS infection without a suspicion of bacterial meningitis. A minimal set of patient characteristics are available for these patients including date of birth, sex, residency, and date that the culture was performed. The first reported date of illness, mostly the first date a culture was taken, was used to calculate age of onset. If not reported (2.6% of patients), the date the isolate was sent to or received by the Reference Laboratory was used.
- PeriNed: The Perinatal Registry has covered approximately 99% of all births in the Netherlands since 2000.^16-18^ It contains data provided by four groups of professionals (e.g. midwives, general practitioners, gynecologists and pediatricians/neonatologists) involved in childbirth care. This data includes variables related to pregnancy, delivery and neonatal (re)admission up until 28 days of age. The registry is managed by PeriNed on behalf of four professional associations: the KNOV (Royal Dutch Organization of Obstetricians), the LHV (National General Practitioners Association), the NVOG (Dutch Association for Obstetrics and Gynecology) and the NVK (Dutch Association for Pediatrics).
- Statistics Netherlands: Statistics Netherlands provides datasets with individual level data to researchers to conduct their own research under strict conditions (For further information: microdata@cbs.nl). Population registry datasets containing variables related to health, well-being, income, and education are linkable using a unique central personal registry number, which is assigned to each Dutch citizen at birth and to residents upon immigration. Population and time periods differ among datasets. All of the results presented in this paper are based on calculations by researchers of Amsterdam UMC and RIVM using the non-public datasets from Statistics Netherlands.
- Dutch Hospital data registry: There are three types of non-public registries available from Statistics Netherlands: hospital admission data (including date of admission and discharge, main diagnosis associated with the hospital admission), diagnosis data (including all diagnoses registered during admission) and data on all care/procedures registered during admission. In the current study, only permission to use these datasets for linkage of patients was obtained, so these datasets were not used for further analyses. Datasets are available from 1995 to 2012.
- Municipal Personal Records Database: This is a national database recording deaths and date of deaths in the Netherlands. The dataset contains information from 1995 to 2019, and is updated every year.
- Dutch Primary School Registry and Dutch Special Education School Registry: This national database on primary school registration and special education was used to identify children in the exposed and unexposed groups who required either enrollment in special schools or additional support in regular schools. Its data cover the period 2008-2019. In the current study, registration in special schools was considered evidence of severe impairment and additional support in standard schools was considered evidence of moderate impairment. To handle missing data in the Netherlands for children who began to be followed in the education database at an older age due to data availability (for example, for a child who was already 8 years old in 2008), we assumed that the outcome at that older age was the same as during preceding years.

# Supplementary Methods

| **Effect modification** | **Effect modification** occurs in biomedical research when a measure of association between an exposure and a health outcome differs according to the levels of a third variable - the effect modifier. The heterogeneity of a measure of association across the levels of an effect modifier helps identifying subgroups for which a risk factor is especially prominent, and specifying a multifactorial causal relationship. Effect modification can be measured on additive and multiplicative scale and literature suggests analysing the presence of the effect modification on both scales (VanderWeele et al, Epidemiol. Methods 2014; Knol et al, International Journal of Epidemiology 2012). There may be a positive modifying effect on the additive scale but a negative or null effect on a multiplicative scale.  The **aim of our study** was to examine whether the effect of iGBS on mortality and long-term NDI differs for boys and girls. As recommended by Knol et al (Knol et al, International Journal of Epidemiology 2012), we performed the following analyses:   1. Stratified analyses by sex, where the risk of outcomes after iGBS were analysed in boys and girls separately; 2. Single reference category analyses using a single reference category of non-iGBS girls; 3. Measures of effect modification on additive scale (by calculating interaction contrasts and RERIs) and on multiplicative scale (by including the product term sex*iGBS in regression models). |
| --- | --- |
|  |  |
| **Mortality analyses** | **Mortality risk, rate, and hazard ratios**: We assessed overall mortality by calculating mortality risk during the first 3 months and the first five years of life and mortality rates per 1000 person-years. Hazard ratios (HRs) and 95% confidence intervals (CIs) were estimated using Cox proportional hazards regression after adjusting for gestational age and birth year. In the stratified analyses by sex, mortality rates among children with a history of iGBS were compared to non-GBS children. In the single reference category analyses, we estimated HRs based on the common reference group of non-iGBS girls.  **Effect modification**: We evaluated the extent to which sex modified the effect of iGBS on overall mortality on both additive and multiplicative scales. Effect modification by sex on the additive scale especially addresses public health-related questions, and effect modification on the multiplicative scale quantifies whether relative associations between iGBS and mortality depend on the child’s sex.  **Additive scale:** Effect modification on the additive scale was examined by calculating the ***interaction contrast***. An interaction contrast It is calculated as the difference between rate differences in sex-specific strata, as follows*:* ***IC = (Mortality rate_iGBS, Boys_ – Mortality rate_non-iGBS, Boys_) – (Mortality rate_IGBS, Girls_ – Mortality rate_non-IGBS, Girls_)***. In addition, we calculated ***attributable proportions*** (= interaction contrast/mortality rate in boys with iGBS).  **Multiplicative scale:** On the multiplicative scale, a modification occurs if relative association measures (i.e., HRs) between exposure and outcome vary by strata of a third variable. We therefore included the product term (sex*iGBS) in multivariable Cox regression models. |
|  |  |
| **NDI analyses** | **NDI and special education risk and odd ratios**: Risks of NDI and need for special education were assessed at the ages of 5 and 10 years. The analyses included only those children followed until at least the corresponding cutoff age. The association between iGBS and NDI was assessed using logistic regression models; estimated odds ratios (ORs) and 95% CIs were adjusted for year of birth and gestational age. In the stratified analyses by sex, NDI risk among children with a history of iGBS were compared to non-GBS children. In the single reference category analyses, we estimated ORs based on the common reference group of non-iGBS girls.  **Effect modification**: We assessed whether sex modified the effect of iGBS on NDI outcomes on both additive and multiplicative scales.  **Additive scale:** Effect modification on the additive scale was examined by calculating the ***relative excess risk due to interaction*** (RERI), using the common reference group of non-iGBS girls and the ORs estimated in the single reference category analyses (***RERI = OR_iGBS, Boys_ – OR_iGBS, Girls_ – OR_non-IGBS, Boys_ + 1***). In addition, we calculated ***attributable proportions*** as RERI/(OR of boys with iGBS).  **Multiplicative scale:** On the multiplicative scale, modification occurs if ORs between iGBS and NDI vary by sex strata. We therefore included the product term (sex*iGBS) in the logistic regression models. |

# Supplementary tables

## Supplementary Table 1A. Effect modification by sex of mortality after GBS sepsis

|  |  | **Single reference category analyses** | | **Stratified analyses** | |
| --- | --- | --- | --- | --- | --- |
|  | **Mortality rate**  **per 1000 PYs** | **HRs (95% CI) for iGBS using a common reference group** | **Effect modification on additive scale**  ***(Interaction contrast [95%CI]); AP [%]*** | **HRs (95% CI) for iGBS within strata of sex** | **Effect modification on multiplicative scale**  ***(p-value of GBS*sex)*** |
| **Denmark** |  |  |  |  |  |
| **0-89d** |  |  |  |  |  |
| GBS-/Girls | 81.20 (65.75–96.66) | *1.00 (reference)* |  | *1.00 (reference)* |  |
| GBS+/Girls | 83.31 (34.08–132.55) | 0.67 (0.36–1.25) |  | 0.69 (0.37–1.28) |  |
| GBS-/Boys | 65.91 (53.70–78.12) | 0.98 (0.75–1.28) |  | *1.00 (reference)* |  |
| GBS+/Boys | 94.00 (47.94–140.06) | 0.98 (0.58–1.66) | *25.98 (-44.26*–*96.22); 27.3%* | 0.98 (0.58–1.66) | *P=0.33* |
| **0-5y** |  |  |  |  |  |
| GBS-/Girls | 4.81 (3.96–5.67) | *1.00 (reference)* |  | *1.00 (reference)* |  |
| GBS+/Girls | 5.11 (2.33–7.88) | 0.70 (0.39–1.24) |  | 0.72 (0.40–1.27) |  |
| GBS-/Boys | 3.94 (3.26–4.62) | 0.97 (0.76–1.25) |  | *1.00 (reference)* |  |
| GBS+/Boys | 4.89 (2.49–7.29) | 0.86 (0.51–1.46) | *0.66 (-3.17*–*4.48); 13.5%* | 0.87 (0.52–1.46) | *P=0.54* |
| **NL** |  |  |  |  |  |
| **0–89d** |  |  |  |  |  |
| GBS-/Girls | 81.6 (57.2–105.9) | *1.00 (reference)* |  | *1.00 (reference)* |  |
| GBS+/Girls | 358.9 (193.1–524.7) | 4.06 (2.34–7.05) |  | 4.06 (2.34–7.04) |  |
| GBS-/Boys | 81.2 (59.5–102.8) | 1.24 (0.83–1.86) |  | *1.00 (reference)* |  |
| GBS+/Boys | 216.2 (103.0–329.5) | 3.06 (1.67–5.59) | *-142.30 (-345.73*–61.14*); NA* | 2.46 (1.37–4.43) | *P=0.22* |
| **0–5y** |  |  |  |  |  |
| GBS-/Girls | 5.3 (3.8–6.8) | *1.00 (reference)* |  | *1.00 (reference)* |  |
| GBS+/Girls | 21.5 (11.6–31.5) | 3.51 (2.04–6.04) |  | 3.49 (2.03–6.02) |  |
| GBS-/Boys | 5.1 (3.8–6.4) | 1.05 (0.71–1.55) |  | *1.00 (reference)* |  |
| GBS+/Boys | 14.5 (7.4–21.7) | 3.18 (1.80–5.61) | *-6.79 (-19.19*–*5.60); NA* | 3.01 (1.73–5.24) | *P=0.71* |

Hazard ratios are adjusted for matching variables (birth year and gestational age)

* due to absence of cases in the extreme preterm age category (<28 weeks), the preterm age categories were merged (<37 weeks) for adjustment purposes

## Supplementary Table 1B. Effect modification by sex of mortality after GBS meningitis

|  |  | **Single reference category analyses** | | **Stratified analyses** | |
| --- | --- | --- | --- | --- | --- |
|  | **Mortality rate**  **per 1000 PYs** | **HRs (95% CI) for iGBS using a common reference group** | **Effect modification on additive scale**  ***(Interaction contrast [95%CI]); AP [%]*** | **HRs (95% CI) for iGBS within strata of sex** | **Effect modification on multiplicative scale**  ***(p-value of GBS*sex)*** |
| **Denmark** |  |  |  |  |  |
| **0-89d** |  |  |  |  |  |
| GBS-/Girls | 28.01 (5.60–50.43) | *1.00 (reference)* |  | *1.00 (reference)* |  |
| GBS+/Girls | 191.16 (3.82–378.49) | 4.62 (1.30–16.49) |  | 5.19 (1.45–18.61) |  |
| GBS-/Boys | 41.94 (12.88–71.01) | 2.10 (0.70–6.31) |  | *1.00 (reference)* |  |
| GBS+/Boys | 161.26 (0.00–343.74) | 5.35 (1.29–22.18) | *-43.83 (-307.9*–*220.25); NA* | 2.39 (0.62–9.25) | *P=0.52* |
| **0-5y** |  |  |  |  |  |
| GBS-/Girls | 2.01 (0.62–3.40) | *1.00 (reference)* |  | *1.00 (reference)* |  |
| GBS+/Girls | 16.17 (3.23–29.11) | 5.43 (1.87–15.76) |  | 6.20 (2.13–18.06) |  |
| GBS-/Boys | 2.24 (0.69–3.79) | 1.42 (0.52–3.88) |  | *1.00 (reference)* |  |
| GBS+/Boys | 8.71 (0.00–18.57) | 3.82 (0.99–14.72) | *-7.69 (-24.09*–*8.71); NA* | 2.39 (0.62–9.25) | *P=0.42* |
| **NL** |  |  |  |  |  |
| **0–89d** |  |  |  |  |  |
| GBS-/Girls | 47.3 (18.0–76.5) | *1.00 (reference)* |  | *1.00 (reference)* |  |
| GBS+/Girls | 295.6 (59.1–532.1) | 5.52 (2.01–15.21) |  | 5.64 (2.05 –15.55) |  |
| GBS-/Boys | 49.1 (22.4–75.7) | 1.21 (0.53–2.79) |  | *1.00 (reference)* |  |
| GBS+/Boys | 531.5 (242.6–820.4) | 11.69 (5.10–26.78) | *234.08 (-141.40*–*609.56); 44.0%* | 9.46 (4.37–20.46) | *P=0.39* |
| **0–5y** |  |  |  |  |  |
| GBS-/Girls | 3.0 (1.2–4.7) | *1.00 (reference)* |  | *1.00 (reference)* |  |
| GBS+/Girls | 17.1 (3.4–30.9) | 5.03 (1.86–13.61) |  | 5.23 (1.93–14.15) |  |
| GBS-/Boys | 3.5 (1.8 –5.1) | 1.92 (0.87–4.25) |  | *1.00 (reference)* |  |
| GBS+/Boys | 29.6 (13.5–45.7) | 14.78 (6.28–34.79) | *11.96 (-9.33*–*33.25); 40.4%* | 8.59 (4.11–17.96) | *P=0.50* |

Hazard ratios are adjusted for matching variables (birth year and gestational age)

* due to absence of cases in the extreme preterm age category (<28 weeks), the preterm age categories were merged (<37 weeks) for adjustment purposes

## Supplementary Table 2A. Effect modification by sex of NDI outcome after GBS sepsis

|  |  | **Single reference category analyses** | | **Stratified analyses** | |
| --- | --- | --- | --- | --- | --- |
|  | **NDI (95% CI)** | **ORs (95% CI) for iGBS using a common reference group** | **Effect modification on**  **additive scale**  ***(RERI [95%CI]); AP [%]*** | **ORs (95% CI) for iGBS within strata of sex** | **Effect modification on multiplicative scale**  ***(p-value of GBS*sex)*** |
| **DENMARK** |  |  |  |  |  |
| **5 years** |  |  |  |  |  |
| **Any** |  |  |  |  |  |
| GBS-/Girls | 1.3% (1.0-17) | *1.00 (reference)* |  | *1.00 (reference)* |  |
| GBS+/Girls | 2.1% (1.0-3.9) | 1.41 (0.71–2.80) |  | 1.43 (0.72–2.84) |  |
| GBS-/Boys | 2.3% (1.9-2.7) | 1.86 (1.37–2.53) |  | *1.00 (reference)* |  |
| GBS+/Boys | 4.6% (3.1-6.6) | 3.63 (2.28–5.79) | *1.36 (-0.35*–*3.08); 37.5%* | 1.95 (1.27–2.99) | *p = 0.43* |
| **Mod-sev** |  |  |  |  |  |
| GBS-/Girls | 0.7% (0.5-1.0) | *1.00 (reference)* |  | *1.00 (reference)* |  |
| GBS+/Girls | 0.8% (0.2-2.2) | 1.07 (0.37–3.07)) |  | 1.05 (0.36–3.05) |  |
| GBS-/Boys | 1.3% (1.1-1.7) | 2.15 (1.41–3.28) |  | *1.00 (reference)* |  |
| GBS+/Boys | 3.2% (2.0-5.0) | 5.02 (2.80–9.00) | *2.80 (0.12*–*5.48); 55.8%* | 2.34 (1.40–3.91) | *p = 0.19* |
| **10 years** |  |  |  |  |  |
| **Any** |  |  |  |  |  |
| GBS-/Girls | 3.0% (2.4-3.6) | *1.00 (reference)* |  | *1.00 (reference)* |  |
| GBS+/Girls | 5.0% (2.9-7.9) | 1.65 (0.97–2.80)) |  | 1.66 (0.97–2.82) |  |
| GBS-/Boys | 7.1% (6.4-7.9) | 2.55 (2.02–3.22) |  | *1.00 (reference)* |  |
| GBS+/Boys | 10.5% (7.9-13.8) | 3.82 (2.65–5.51) | *0.62 (-0.83*–*2.07); 16.2%* | 1.50 (1.08–2.07) | *p = 0.76* |
| **Mod-sev** |  |  |  |  |  |
| GBS-/Girls | 1.2% (0.9-1.7) | *1.00 (reference)* |  | *1.00 (reference)* |  |
| GBS+/Girls | 2.1% (0.8-4.2) | 1.59 (0.70–3.58) |  | 1.61 (0.71–3.63) |  |
| GBS-/Boys | 3.1% (2.6-3.7) | 2.63 (1.85–3.74) |  | *1.00 (reference)* |  |
| GBS+/Boys | 4.7% (2.9-7.1) | 3.90 (2.28–6.68) | *0.69 (-1.46*–*2.83); 17.7%* | 1.48 (0.92–2.38) | *p = 0.89* |
| **NL** |  |  |  |  |  |
| **5 years** |  |  |  |  |  |
| **Any** |  |  |  |  |  |
| GBS-/Girls | 0.7% (0.4-1.4) | *1.00 (reference)* |  | *1.00 (reference)* |  |
| GBS+/Girls | 3.5% (1.2-8.0) | 4.89 (1.63–14.68) |  | 4.86 (1.58–14.95) |  |
| GBS-/Boys | 1.3% (0.8-1.9) | 2.07 (0.98–4.39) |  | *1.00 (reference)* |  |
| GBS+/Boys | 2.1% (0.6-5.2) | 3.29 (1.01–10.74) | *-2.68 (-8.70–3.35); NA* | 1.63 (0.56–4.77) | *p = 0.15* |
| **Mod-sev** |  |  |  |  |  |
| GBS-/Girls | 0.7% (0.4-1.4) | *1.00 (reference)* |  | *1.00 (reference)* |  |
| GBS+/Girls | 2.8% (0.8-7.1) | 3.83 (1.17–12.56) |  | 3.80 (1.13–12.81) |  |
| GBS-/Boys | 0.6% (0.3-1.1) | 1.06 (0.45–2.50) |  | *1.00 (reference)* |  |
| GBS+/Boys | 2.1% (0.6-5.2) | 3.37 (1.02–11.12) | *-0.51 (-5.79–4.76); NA* | 3.25 (1.03–10.23) | *p = 0.83* |
| **10 years** |  |  |  |  |  |
| **Any** |  |  |  |  |  |
| GBS-/Girls | 2.5% (1.5-4.0) | *1.00 (reference)* |  | *1.00 (reference)* |  |
| GBS+/Girls | 4.1% (0.9-11.5) | 1.60 (0.46–5.60) |  | 1.56 (0.44–5.56) |  |
| GBS-/Boys | 8.8% (7.1-10.8) | 3.97 (2.36–6.69) |  | *1.00 (reference)* |  |
| GBS+/Boys | 18.8% (11.5-28.0) | 9.50 (4.71–19.15) | *4.93 (-0.73–10.59); 51.9%* | 2.39 (1.36–4.20) | *p = 0.57* |
| **Mod-sev** |  |  |  |  |  |
| GBS-/Girls | 0.7 (0.2-1.6) | *1.00 (reference)* |  | *1.00 (reference)* |  |
| GBS+/Girls | 4.1% (0.9-11.5) | 6.21 (1.45–26.67) |  | 7.04 (1.53–32.43) |  |
| GBS-/Boys | 4.9% (3.6-6.5) | 7.57 (2.99–19.19) |  | *1.00 (reference)* |  |
| GBS+/Boys | 10.4% (5.1-18.3) | 17.64 (5.85–53.15) | *4.86 (-9.47–19.18); 27.6%* | 2.32 (1.13–4.76) | *p = 0.24* |

Odds ratio are adjusted for matching variables (birth months/year and gestational age);

* due to absence of cases in the extreme preterm age category (<28 weeks), the preterm age categories were merged (<37 weeks) for adjustment purposes.

## Supplementary Table 2B. Effect modification by sex of NDI outcome after GBS meningitis

|  |  | **Single reference category analyses** | | **Stratified analyses** | |
| --- | --- | --- | --- | --- | --- |
|  | **NDI (95% CI)** | **ORs (95% CI) for iGBS using a common reference group** | **Effect modification on**  **additive scale**  ***(RERI [95%CI]); AP [%]*** | **ORs (95% CI) for iGBS within strata of sex** | **Effect modification on multiplicative scale**  ***(p-value of GBS*sex)*** |
| **DENMARK** |  |  |  |  |  |
| **5 years** |  |  |  |  |  |
| **Any** |  |  |  |  |  |
| GBS-/Girls | 2.1% (1.2-3.5) | *1.00 (reference)* |  | *1.00 (reference)* |  |
| GBS+/Girls | 14.3% (6.8-25.4) | 7.59 (3.10–18.58) |  | 7.88 (3.18–19.51) |  |
| GBS-/Boys | 1.7% (0.8-3.1) | 0.76 (0.33–1.74) |  | *1.00 (reference)* |  |
| GBS+/Boys | 16.9% (8.4-29.0) | 9.27 (3.84–22.34) | *1.92 (-6.61-10.45); 20.7%* | 11.88 (4.64–30.39) | *p = 0.48* |
| **Mod-sev** |  |  |  |  |  |
| GBS-/Girls | 1.7% (0.8-3.0) | *1.00 (reference)* |  | *1.00 (reference)* |  |
| GBS+/Girls | 9.5% (3.6-19.6) | 6.04 (2.11–17.28) |  | 6.28 (2.17–18.13) |  |
| GBS-/Boys | 0.9% (0.3-2.0) | 0.47 (0.16–1.37) |  | *1.00 (reference)* |  |
| GBS+/Boys | 13.6% (6.0-25.0) | 8.75 (3.29–23.27) | *3.24 (-7.92-5.60); 37.0%* | 19.26 (5.86–63.31) | *p = 0.15* |
| **10 years** |  |  |  |  |  |
| **Any** |  |  |  |  |  |
| GBS-/Girls | 4.4% (2.8-6.7) | *1.00 (reference)* |  | *1.00 (reference)* |  |
| GBS+/Girls | 23.9% (12.6-38.8) | 6.88 (3.03–15.61) |  | 6.86 (3.03–15.57) |  |
| GBS-/Boys | 9.1% (6.4-12.4) | 1.96 (1.11–3.45) |  | *1.00 (reference)* |  |
| GBS+/Boys | 22.5% (10.8-38.5) | 5.71 (2.38–13.69) | *-2.13 (-8.73*–*4.47); NA* | 2.93 (1.27–6.77) | *p = 0.18* |
| **Mod-sev** |  |  |  |  |  |
| GBS-/Girls | 3.0% (1.6-4.9) | *1.00 (reference)* |  | *1.00 (reference)* |  |
| GBS+/Girls | 15.2% (6.3-28.9) | 5.85 (2.20–15.59) |  | 5.93 (2.22–15.87) |  |
| GBS-/Boys | 4.7% (2.8-7.3) | 1.45 (0.71–2.99) |  | *1.00 (reference)* |  |
| GBS+/Boys | 15.0% (5.7-29.8) | 5.15 (1.83–14.49) | ***-****1.16 (-7.92*–*5.60); NA* | 3.55 (1.30–9.74) | *p = 0.48* |
| **NL** |  |  |  |  |  |
| **5 years** |  |  |  |  |  |
| **Any** |  |  |  |  |  |
| GBS-/Girls | 0.5% (0.1-1.5) | *1.00 (reference)* |  | *1.00 (reference)* |  |
| GBS+/Girls | 3.4% (0.4-11.7) | 6.55 (1.05–40.72) |  | 7.35 (1.09–49.42) |  |
| GBS-/Boys | 1.9% (1.1-3.1) | 4.06 (1.16–14.26) |  | *1.00 (reference)* |  |
| GBS+/Boys | 10.1% (4.5-19.0) | 24.33 (6.15–96.33) | *14.72 (-11.53-40.96); 60.5%* | 5.98 (2.39–14.96) | *p = 0.93* |
| **Mod-sev** |  |  |  |  |  |
| GBS-/Girls | 0.3% (0.04-1.2) | *1.00 (reference)* |  | *1.00 (reference)* |  |
| GBS+/Girls | 3.4% (0.4-11.7) | 10.01 (1.36–73.47) |  | 11.84 (1.43–98.41) |  |
| GBS-/Boys | 1.7% (0.9-2.8) | 5.00 (1.11–22.44) |  | *1.00 (reference)* |  |
| GBS+/Boys | 7.6% (2.8-15.8) | 24.86 (4.84–127.75) | *10.84 (-18.48-40.16); 43.6%* | 4.90 (1.78–13.46) | *p = 0.87* |
| **10 years** |  |  |  |  |  |
| **Any** |  |  |  |  |  |
| GBS-/Girls | 3.3% (1.6-5.8) | *1.00 (reference)* |  | *1.00 (reference)* |  |
| GBS+/Girls | 9.4% (2.0-25.0) | 3.02 (0.79–11.56) |  | 3.05 (0.80–11.71) |  |
| GBS-/Boys | 8.3% (6.1-11.0) | 2.74 (1.39–5.42) |  | *1.00 (reference)* |  |
| GBS+/Boys | 23.5% (12.8-37.5) | 9.43 (3.87–22.99) | *4.66 (-2.89-12.20); 49.4%* | 3.43 (1.66–7.08) | *p = 0.54* |
| **Mod-sev** |  |  |  |  |  |
| GBS-/Girls | 1.8% (0.7-3.8) | *1.00 (reference)* |  | *1.00 (reference)* |  |
| GBS+/Girls | 3.1% (0.8-16.2) | 1.73 (0.20–15.00) |  | 1.73 (0.20–15.15) |  |
| GBS-/Boys | 5.2% (3.5-7.5) | 3.16 (1.29–7.79) |  | *1.00 (reference)* |  |
| GBS+/Boys | 17.6% (8.4-30.9) | 12.61 (4.21–37.74) | *8.71 (-3.08-20.51); 69.1%* | 3.96 (1.73–9.09) | *p = 0.48* |

Odds ratio are adjusted for matching variables (birth year and gestational age);

* due to absence of cases in the extreme preterm age category (<28 weeks), the preterm age categories were merged (<37 weeks) for adjustment purposes.

Supplementary Table 3. Effect modification by sex in analysis that used non-gestational age matched comparison cohort

For this analysis, unexposed children (i.e., children with no history of invasive GBS disease) were matched only on year and month of birth and sex to exposed children included in the primary analyses.

(A) Non-gestational age-matched analysis: Effect modification by sex of the association between invasive GBS disease and mortality. Hazard ratios are adjusted for birth year

|  |  | **Single reference category analyses** | | **Stratified analyses** | |
| --- | --- | --- | --- | --- | --- |
|  | **Mortality rate**  **per 1000 PYs** | **HRs (95% CI) for iGBS using a common reference group** | **Effect modification on additive scale**  ***(Interaction contrast [95%CI] and AP)*** | **HRs (95% CI) for iGBS within strata of sex** | **Effect modification on multiplicative scale**  ***(p-value of GBS*sex)*** |
| **Denmark*** |  |  |  |  |  |
| **0-5y** |  |  |  |  |  |
| GBS-/Girls | 0.7 (0.4–1.0) | *1.00 (reference)* |  | *1.00 (reference)* |  |
| GBS+/Girls | 6.5 (3.6–9.4) | 8.76 (4.74-16.18) |  | 8.78 (4.75-16.21) |  |
| GBS-/Boys | 1.3 (0.9–1.6) | 1.70 (1.02–2.83) |  | *1.00 (reference)* |  |
| GBS+/Boys | 5.3 (2.9–7.6) | 7.11 (3.84–13.13) | *-1.8 (-5.6;2.0); NA* | 4.18 (2.45–7.13) | *P=0.07* |
| **NL**** |  |  |  |  |  |
| **0–5y** |  |  |  |  |  |
| GBS-/Girls | 1.1 (0.2–1.7) | *1.00 (reference)* |  | *1.00 (reference)* |  |
| GBS+/Girls | 20.2 (12–1-28.3) | 16.8 (8.70–32.51) |  | 16.85 (8.72–32.56) |  |
| GBS-/Boys | 1.6 (1.0–1.2) | 1.4 (0.75–2.74) |  | *1.00 (reference)* |  |
| GBS+/Boys | 18.8 (12.0–25.7) | 15.6 (8.25–29.55) | *-1.9 (-2.5;-1.3); NA* | 10.89 (6.41–18.49) | *P=0.32* |

(B) Non-gestational age-matched analysis: Effect modification by sex of the association between invasive GBS disease and neurodevelopmental impairment. Odds ratios are adjusted for birth year

|  |  | **Single reference category analyses** | | **Stratified analyses** | |
| --- | --- | --- | --- | --- | --- |
|  | **NDI** | **ORs (95% CI) for iGBS using a common reference group** | **Effect modification on**  **additive scale**  ***(RERI [95%CI] and AP)*** | **ORs (95% CI) for iGBS within strata of sex** | **Effect modification on multiplicative scale**  ***(p-value of GBS*sex)*** |
| **DENMARK** |  |  |  |  |  |
| **5 years** |  |  |  |  |  |
| **Any** |  |  |  |  |  |
| GBS-/Girls | 1.0% | *1.00 (reference)* |  | *1.00 (reference)* |  |
| GBS+/Girls | 3.5% | 3.57 (2.11–6.07) |  | 3.58 (2.11–6.08) |  |
| GBS-/Boys | 2.0% | 1.95 (1.42–2.68) |  | *1.00 (reference)* |  |
| GBS+/Boys | 5.7% | 5.93 (3.88–9.08) | *1.41 (-1.18*; 3*.99); 23.8%* | 3.05 (2.09–4.44) | *p = 0.63* |
| **Mod-sev** |  |  |  |  |  |
| GBS-/Girls | 0.5% | *1.00 (reference)* |  | *1.00 (reference)* |  |
| GBS+/Girls | 1.9% | 3.53 (1.71–7.27) |  | 3.53 (1.71–7.23) |  |
| GBS-/Boys | 1.1% | 2.16 (1.40–3.33) |  | *1.00 (reference)* |  |
| GBS+/Boys | 4.2% | 8.18 (4.81–13.91) | *3.49 (-0.49;7.48); 42.7%* | 3.79 (2.42–5.94) | *p = 0.87* |
| **NL** |  |  |  |  |  |
| **5 years** |  |  |  |  |  |
| **Any** |  |  |  |  |  |
| GBS-/Girls | 0.3% | *1.00 (reference)* |  | *1.00 (reference)* |  |
| GBS+/Girls | 3.5% | 11.62 (3.86–34.98) |  | 11.65 (3.86-35.13) |  |
| GBS-/Boys | 1.4% | 4.83 (2.04–11.46) |  | *1.00 (reference)* |  |
| GBS+/Boys | 4.4% | 15.29 (5.68–41.18) | -0.16 (-12.79-12.48); N.A. | 3.16 (1.63-6.14) | *p = 0.048* |
| **Mod-sev** |  |  |  |  |  |
| GBS-/Girls | 0.2% | *1.00 (reference)* |  | *1.00 (reference)* |  |
| GBS+/Girls | 3.0% | 14.84 (4.15-53.06) |  | 14.87 (4.15-53.26) |  |
| GBS-/Boys | 1.0% | 4.70 (1.63-13.54) |  | *1.00 (reference)* |  |
| GBS+/Boys | 3.7% | 18.72 (5.83-60.17) | 0.18 (-16.85-17.21); 1.0% | 3.98 (1.89-8.38) | *p = 0.08* |

## Supplementary Table 4. Effect modification by sex of domain-specific NDI outcomes after invasive GBS disease in Denmark

|  | **NDI** | **OR (95% CI)** | **NDI** | **OR (95% CI)** | **ORs (95% CI) for iGBS within strata of sex** |
| --- | --- | --- | --- | --- | --- |
| **5 yr** | **GBS-** |  | **GBS+** |  |  |
| **Visual** |  |  |  |  |  |
| Girls | 0.2% | 1.0 (*reference*) | 0.7% | 3.52 (1.09–11.37) | 4.06 (1.27–12.99) |
| Boys | 0.3% | 1.79 (0.84–3.81) | 0.6% | 3.19 (0.99–10.22) | 1.67 (0.56–4.95) |
|  |  |  |  | ***RERI (95%CI)*** | ***GBS*sex term*** |
|  |  |  |  | *-1.12 (-6.06*–*3.82)* | *p = 0.40* |
| **Hearing** |  |  |  |  |  |
| Girls | 0.1% | 1.0 (*reference*) | 0.7% | 6.77 (1.90–24.09) | 6.76 (1.90–24.07) |
| Boys | 0.3% | 2.90 (1.17–7.21) | 0.5% | 4.16 (1.04–16.68) | 1.43 (0.43–4.81) |
|  |  |  |  | ***RERI (95%CI)*** | ***GBS*sex term*** |
|  |  |  |  | *-4.52 (-13.89–4.85)* | *p = 0.08* |
| **Cognitive** |  |  |  |  |  |
| Girls | 0.5% | 1.0 (*reference*) | 1.1% | 2.03 (0.84–4.94) | 2.04 (0.83–4.96) |
| Boys | 1.2% | 2.23 (1.44–3.45) | 2.2% | 4.14 (2.17–7.92) | 1.86 (1.04–3.32) |
|  |  |  |  | ***RERI (95%CI)*** | ***GBS*sex term*** |
|  |  |  |  | *0.89 (-1.90–3.67)* | *p = 0.87* |
| **Social** |  |  |  |  |  |
| Girls | 0.5% | 1.0 (*reference*) | 1.3% | 2.50 (1.07–5.86) | 2.51 (1.07–5.90) |
| Boys | 0.7% | 1.48 (0.91–2.43) | 1.5% | 3.20 (1.53–6.72) | 2.16 (1.08–4.33) |
|  |  |  |  | ***RERI (95%CI)*** | ***GBS*sex term*** |
|  |  |  |  | *0.22 (-2.59–3.02)* | *p = 0.79* |
| **Motor** |  |  |  |  |  |
| Girls | 0.5% | 1.0 (*reference*) | 1.5% | 2.51 (1.12–5.62) | 2.55 (1.13–5.75) |
| Boys | 0.5% | 1.09 (0.65–1.82) | 3.2% | 6.56 (3.65–11.78) | 5.98 (3.42–10.47) |
|  |  |  |  | ***RERI (95%CI)*** | ***GBS*sex term*** |
|  |  |  |  | *3.96 (0.33–7.59)* | *p = 0.08* |
| **10 yr** | **GBS-** |  | **GBS+** |  |  |
| **Visual** |  |  |  |  |  |
| Girls | 0.2% | 1.0 (*reference*) | 1.6% | 6.13 (2.16–17.43) | 6.40 (2.25–18.18) |
| Boys | 0.6% | 2.42 (1.14–5.16) | 0.6% | 2.49 (0.67–9.25) | 1.00 (0.30–3.33) |
|  |  |  |  | ***RERI (95%CI)*** | ***GBS*sex term*** |
|  |  |  |  | *-5.06 (-12.09–1.96)* | *p = 0.03* |
| **Hearing** |  |  |  |  |  |
| Girls | 0.3% | 1.0 (*reference*) | 1.0% | 3.09 (1.00–9.52) | 3.09 (1.00–9.54) |
| Boys | 0.6% | 1.63 (0.84–3.17) | 1.2% | 3.63 (1.37–9.61) | 2.23 (0.91–5.42) |
|  |  |  |  | ***RERI (95%CI)*** | ***GBS*sex term*** |
|  |  |  |  | *-0.09 (-4.39–4.22)* | *p = 0.66* |
| **Cognitive** |  |  |  |  |  |
| Girls | 1.5% | 1.0 (*reference*) | 2.6% | 1.71 (0.86–3.38) | 1.72 (0.87–3.40) |
| Boys | 3.7% | 2.48 (1.84–3.36) | 4.7% | 3.21 (1.96–5.26) | 1.29 (0.83–2.01) |
|  |  |  |  | ***RERI (95%CI)*** | ***GBS*sex term*** |
|  |  |  |  | *0.02 (-1.76–1.80)* | *p = 0.50* |
| **Social** |  |  |  |  |  |
| Girls | 1.7% | 1.0 (*reference*) | 3.1% | 1.79 (0.96–3.34) | 1.77 (0.95–3.32) |
| Boys | 4.7% | 2.82 (2.13–3.74) | 7.2% | 4.38 (2.87–6.69) | 1.56 (1.07–2.25) |
|  |  |  |  | ***RERI (95%CI)*** | ***GBS*sex term*** |
|  |  |  |  | *0.77 (-1.10–2.64)* | *p = 0.70* |
| **Motor** |  |  |  |  |  |
| Girls | 0.7% | 1.0 (*reference*) | 2.1% | 2.90 (1.28–6.57) | 2.96 (1.31–6.72) |
| Boys | 0.8% | 1.20 (0.72–2.00) | 2.9% | 4.29 (2.20–8.39) | 3.54 (1.88–6.68) |
|  |  |  |  | ***RERI (95%CI)*** | ***GBS*sex term*** |
|  |  |  |  | *1.19 (-1.95–4.32)* | *p = 0.69* |

Supplementary Figure 1. Proportion of children with domain-specific neurodevelopmental impairments in Denmark for boys and girls with and without history of iGBS by the ages of 5 (A) and 10 years (B).


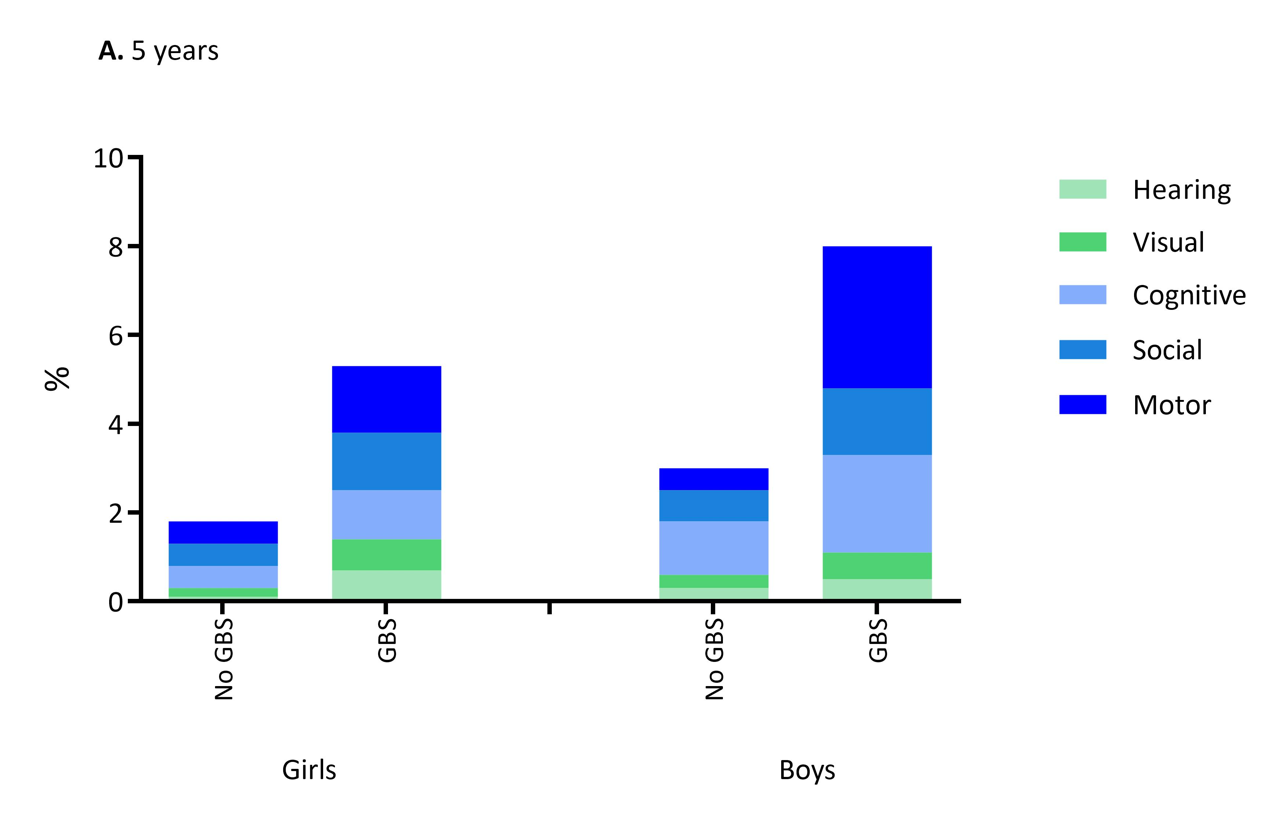

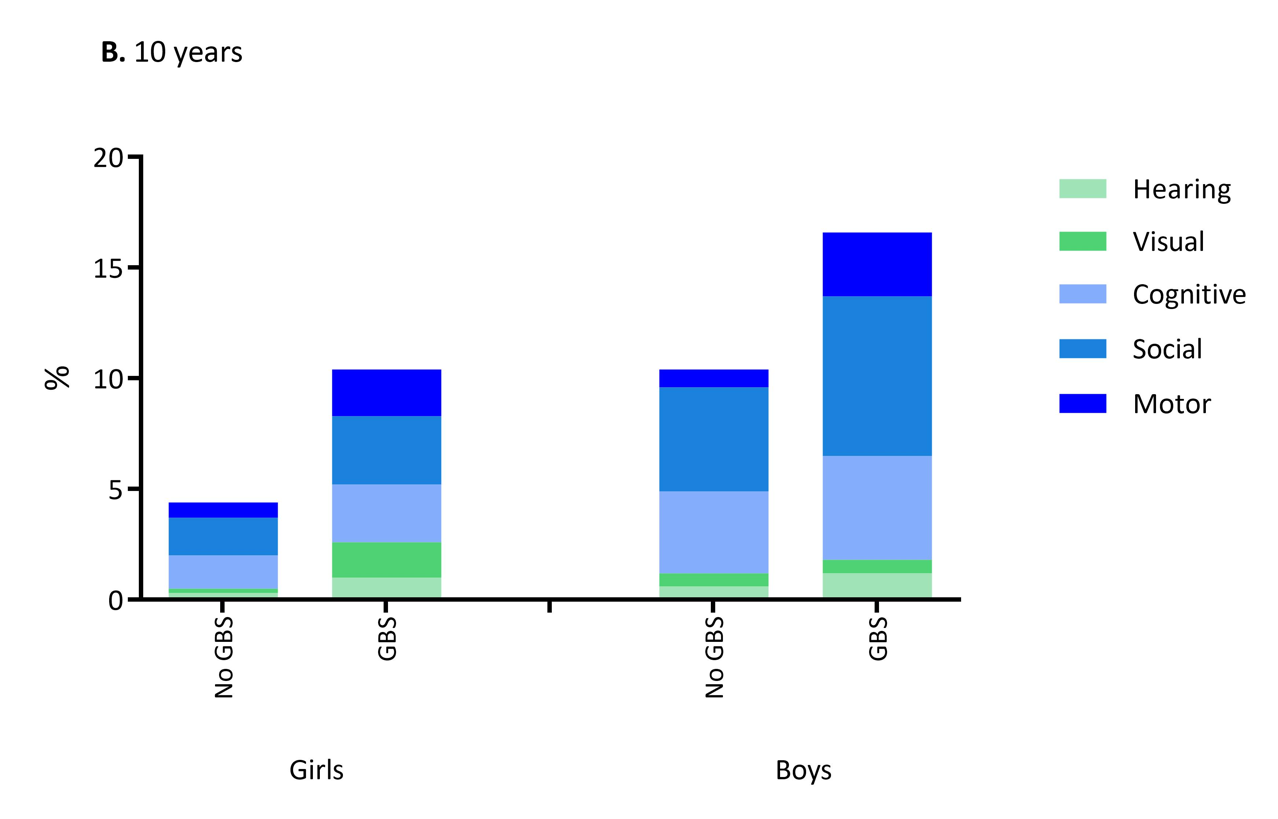


# References

1. Horvath-Puho E, van Kassel MN, Goncalves BP, et al. Mortality, neurodevelopmental impairments, and economic outcomes after invasive group B streptococcal disease in early infancy in Denmark and the Netherlands: a national matched cohort study. *Lancet Child Adolesc Health* 2021.

2. Schmidt M, Schmidt SAJ, Adelborg K, et al. The Danish health care system and epidemiological research: from health care contacts to database records. *Clin Epidemiol* 2019; **11**: 563-91.

3. Schmidt M, Pedersen L, Sorensen HT. The Danish Civil Registration System as a tool in epidemiology. *Eur J Epidemiol* 2014; **29**(8): 541-9.

4. Bliddal M, Broe A, Pottegard A, Olsen J, Langhoff-Roos J. The Danish Medical Birth Register. *Eur J Epidemiol* 2018; **33**(1): 27-36.

5. Cohen E, Horvath-Puho E, Ray JG, et al. Association Between the Birth of an Infant With Major Congenital Anomalies and Subsequent Risk of Mortality in Their Mothers. *JAMA* 2016; **316**(23): 2515-24.

6. Sorensen HT, Horvath-Puho E, Pedersen L, Baron JA, Prandoni P. Venous thromboembolism and subsequent hospitalisation due to acute arterial cardiovascular events: a 20-year cohort study. *Lancet* 2007; **370**(9601): 1773-9.

7. Sundboll J, Horvath-Puho E, Adelborg K, et al. Higher Risk of Vascular Dementia in Myocardial Infarction Survivors. *Circulation* 2018; **137**(6): 567-77.

8. Schmidt M, Schmidt SA, Sandegaard JL, Ehrenstein V, Pedersen L, Sorensen HT. The Danish National Patient Registry: a review of content, data quality, and research potential. *Clin Epidemiol* 2015; **7**: 449-90.

9. Mors O, Perto GP, Mortensen PB. The Danish Psychiatric Central Research Register. *Scand J Public Health* 2011; **39**(7 Suppl): 54-7.

10. Baadsgaard M, Quitzau J. Danish registers on personal income and transfer payments. *Scand J Public Health* 2011; **39**(7 Suppl): 103-5.

11. World Bank national accounts data, and OECD National Accounts data files. Economic Policy & Debt: National accounts: Shares of GDP & other; 2020.

12. European Central Bank Statistical Data Warehouse. 2020.

13. Faber MJ, Burgers JS, Westert GP. A sustainable primary care system: lessons from the Netherlands. *J Ambul Care Manage* 2012; **35**(3): 174-81.

14. Netherlands Reference Laboratory For Bacterial Meningitis (AMC/RIVM). Bacterial meningitis in The Netherlands: Annual Report 2016. Amsterdam: University of Amsterdam; 2017.

15. Bijlsma MW, Bekker V, Brouwer MC, Spanjaard L, van de Beek D, van der Ende A. Epidemiology of invasive meningococcal disease in the Netherlands, 1960-2012: an analysis of national surveillance data. *Lancet Infect Dis* 2014; **14**(9): 805-12.

16. Statistics Netherlands. StatLine, The Hague/Heerlen, 2017 (Assessed October 24, 2017, at <http://www.cbs.nl>)

17. Eskes M, Ensing S, Groenendaal F, Abu-Hanna A, Ravelli A. The risk of intrapartum/neonatal mortality and morbidity following birth at 37 weeks of gestation: a nationwide cohort study. *BJOG* 2019; **126**(10): 1252-7.

18. PeriNed. Perinatale Zorg in Nederland (2000-2017) [Annual reports (2000-2017) PeriNed]. Utrecht; 2019.
